# Effects of natural and artificial shade on behavior, physiology, and productivity in water buffalo: a narrative review

**DOI:** 10.3389/fvets.2026.1730075

**Published:** 2026-03-11

**Authors:** Daniel Mota-Rojas, Lydia Lanzoni, Arthur Fernandes Bettencourt, Adriana Domínguez-Oliva, Alfonso Chay-Canul, Adolfo Álvarez-Macías, Andrea Bragaglio, Ismael Hernández-Avalos, Vivian Fischer, Eleonora Nannoni, Ayman H. Abd El-Aziz, Patricia Mora-Medina, Julio Martínez-Burnes, Adriana Olmos-Hernández, Fabiola Torres-Bernal, Nancy José-Pérez, Vittoria Lucia Barile

**Affiliations:** 1 Neurophysiology, Behavior and Animal Welfare Assessment, DPAA, Universidad Autónoma Metropolitana (UAM), Mexico City, Mexico; 2 Animal Production and Health Division, Food and Agriculture Organization (FAO), Rome, Italy; 3 Department of Animal Science, Federal University of Santa Maria, Santa Maria, Rio Grande do Sul, Brazil; 4 División Académica de Ciencias Agropecuarias, Universidad Juárez Autónoma de Tabasco, Villahermosa, Mexico; 5 CREA Research Centre for Engineering and Agro-Food Processing, Consiglio per la Ricerca in Agricoltura el'Analisi dell'Economia Agraria, Treviglio, Italy; 6 Exo Research Organization, Potenza, Italy; 7 Facultad de Estudios Superiores Cuautitlán, FESC, Universidad Nacional Autónoma de México (UNAM), Cuautitlán Izcalli, State of Mexico, Mexico; 8 Department of Animal Science, Federal University of Rio Grande do Sul, Porto Alegre, Rio Grande do Sul, Brazil; 9 Department of Veterinary Medical Sciences, DIMEVET, University of Bologna, Bologna, Italy; 10 Animal Husbandry and Animal Wealth Development Department, Faculty of Veterinary Medicine, Damanhour University, Damanhour, Egypt; 11 Instituto de Ecología Aplicada, Facultad de Medicina Veterinaria y Zootecnia, Universidad Autónoma de Tamaulipas, Ciudad Victoria, Mexico; 12 Division of Biotechnology, Instituto Nacional de Rehabilitación Luis Guillermo Ibarra Ibarra (INR-LGII), Mexico City, Mexico; 13 Council for Agricultural Research and Economics (CREA)—Research Centre for Animal Production and Aquaculture, Monterotondo, Italy; 14 International Buffalo Federation (IBF), Monterotondo, Italy

**Keywords:** *Bubalus bubalis*, roof, shade provision, silvopastoral systems, tree shade

## Abstract

Although water buffaloes (*Bubalus bubalis*) are a species known for their adaptability to different environments, their anatomical characteristics make them susceptible to heat stress in hot-humid climate conditions. In productive systems, the provision of natural or artificial shade is an alternative to mitigate the negative effects of heat stress and maintain the animals' thermal balance. However, most of the research addressing shade provision is focused on *Bos taurus* and *Bos indicus* species. Thus, the present review aims to discuss the effects of providing natural or artificial shade on physiological, behavioral, and productive parameters of water buffalo as a management strategy to mitigate thermal stress. Some studies in cattle will be used to compare and analyze the effect of shade on animal welfare.

## 1 Introduction

**Figure 1:**
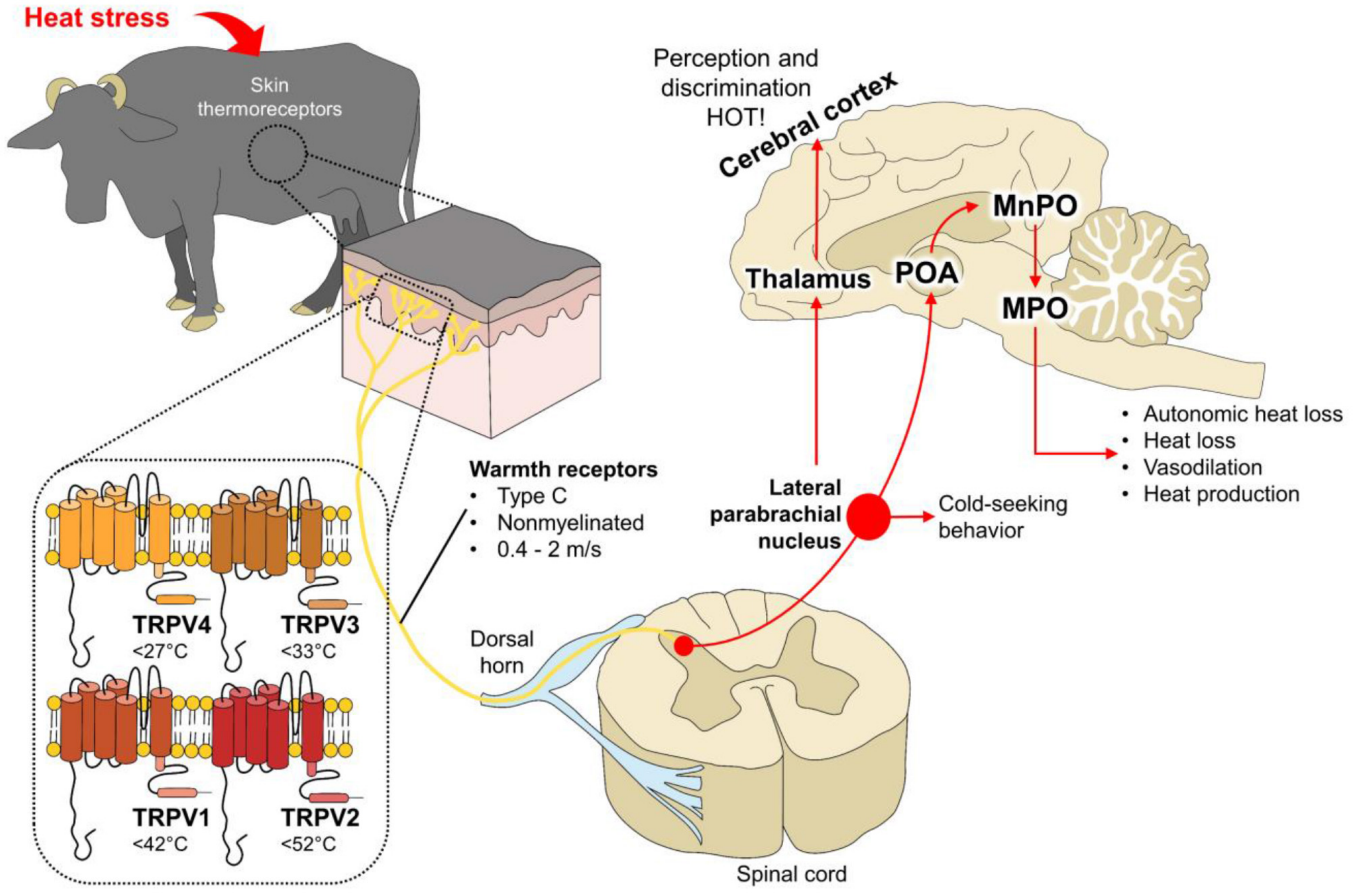
Heat stress, peripheral thermoreceptors, and thermoregulatory mechanisms in water buffalo.

Water buffaloes (*Bubalus bubalis*) are a species adapted to production systems in hot and humid environments. However, increasingly hot climate conditions–mainly due to climate change–can cause physiological and behavioral alterations due to their thermal tolerance range (e.g., 25–26 °C is considered the upper limit temperature for dairy cattle) (1–3). Heat stress (HS) is a state in which animals cannot efficiently dissipate excess heat. It is a gradient of increasing effort to adapt to ambient temperature above the thermoneutral range of the animal and is directly related to environmental conditions. HS compromises animal welfare by eliciting physiological alterations such as increased respiratory rate (RR), dehydration, and acid-base imbalance (Figure 1) (4, 5). Although water buffaloes are mainly raised in hot climates, they can be susceptible to HS due to anatomical characteristics, such as their dark skin (0.407 ± 0.306 μg/mg melanocytes), low sweat gland density (394 glands/cm^2^), and thick epidermis (up to 115 μm), which impairs their ability to dissipate heat (6, 7). The low evaporative efficiency and higher absorption of solar radiation of water buffalo suggest the need for a differentiated evaluation from *B. taurus* and *B. indicus*. In contrast to dairy cattle, information on the adaptation mechanisms of water buffalo is limited. Standard thermal limits for dairy cattle may be inadequate for buffaloes, considering that they have different thermoregulatory efficiency.

Environments with high temperatures induce behavioral changes, including reduced activity, decreased feed intake (8), shade seeking (9, 10), and wallowing (7, 11). Shade-seeking behavior is one of the primary strategies of ruminants to mitigate HS; thus, providing natural or artificial shade to animals raised in extensive and intensive production systems is considered an alternative to prevent HS-related alterations (12, 13).

When exposed to hot environmental temperatures, thermoreceptors such as the Transient Receptor Potential Vanilloid (TRPV4 or TRPV3) initiate the physiological cascade to thermoregulate. Thermal information is transmitted through warmth receptors to the dorsal horn of the spinal cord. Subsequently, information is projected to the lateral parabrachial nucleus and directly to the preoptic area of the hypothalamus (POA), the thermoregulatory center. Through connections to the median preoptic area (MnPO), and medial preoptic area (MPOA), several autonomic responses to promote heat loss are elicited. Moreover, activation of the lateral parabrachial nucleus initiates cold-seeking behaviors such as shade seeking to reduce body temperature.

Natural shade, provided mainly by trees or vegetation, offers multiple advantages by reducing solar radiation and ambient temperature in extensive or silvopastoral systems (SPS). Several studies have documented that buffaloes prefer to remain in shaded areas during hours of greatest radiation, and access to natural shade reduces physiological changes due to HS. For example, shade-seeking behavior was encouraged in Nili Ravi buffaloes during events with a high temperature-humidity index (THI) (82.1), in contrast to lower THI (77.8) (14). This climate index, which integrates temperature and humidity to assess HS, determines the animal's comfort threshold. Providing natural shade also influences the physiological response of ruminants. This was reported in female Murrah buffaloes, in whom parameters such as RR (26.6 mov/min), excessive panting, dorsal flank surface temperature (1.6 °C), and rectal temperature (RT; 0.86 °C less) were significantly reduced when they had access to the shade of red Jambeiro trees, even at hours (14:00) with high solar radiation (168.57 KJ/m^2^) (15). Moreover, a reduction in water-seeking behavior, has also been reported (16).

Artificial shade, on the other hand, constitutes a strategic alternative in production systems where vegetation cover is scarce or non-existent, and in intensive facilities (17). A study performed in India, in the Namakkal district, found that out of 120 farmers, 92% provided artificial shelters to the buffaloes, and 75% used thatched roof sheds (18). Roof material greatly differs; however, as mentioned by Özdemir (19), 80% of roofing material is sheet metal/Eternit (fiber-cement) in Turkey provinces (the second-highest buffalo producer with 22% of milk yield and around 800–900 L annually) (20, 21). The roof material directly influences the animal's thermoregulation, an aspect that has been mainly valued in dairy cattle. In *Bos taurus* and *B. indicus* crossbreed cattle, the provision of artificial shade reduced RT (0.7 °C), RR (8.1 breaths/min), body surface temperature (mean of 1.4 °C), and plasma cortisol levels (0.3 ng/ml mean), in addition to lowering the panting score to 1.3 (10). In the case of water buffalo, Gu et al. (9) found that the provision of a shade shelter with 99% blockage of solar radiation in the loafing area decreased the RR from 31.6 to 25.3 breaths/min in Dehong buffalo calves. Moreover, behavioral changes are also observed as increased water intake and decreased feeding or grazing (22–24), which might affect the productive performance in buffalo farming systems.

Whether natural or artificial, providing shaded areas for water buffaloes prevents adverse effects of direct solar radiation, including high body temperature, tachypnea, and elevated body surface temperature (15, 25–27). Not providing shade predisposes animals to HS, which decreases feed intake and, consequently, milk yield (28). Mastitis, metritis, and cases of retained placenta and infertility issues have also been associated with HS (28). Comparative analysis between natural and artificial shade should not be limited solely to the immediate physiological response but should also consider other variables, such as milk yield, feed efficiency, weight gain, and reproductive indicators. Evidence on the influence of natural and artificial shade has been extensively evaluated in *B. taurus* and *B. indicus*; however, studies on *B. bubalis* are limited. Furthermore, buffaloes present unique anatomical and behavioral traits that may lead to different responses to shade when compared to cattle, reinforcing the need for species-specific evaluations. In addition, little is known about the potential effects of shade on social behavior, metabolic parameters, or long-term welfare outcomes in buffaloes. In this sense, this review aims to discuss the effects of providing natural or artificial shade on physiological, behavioral, and productive parameters of water buffalo as a management strategy to mitigate HS. The study will also address the negative effects of HS and high ambient temperatures and how providing shade diminishes these effects. Through the manuscript, a comparative discussion between studies on *B. bubalis* and *Bos taurus/Bos indicus* will be used when no available studies have been performed in water buffaloes.

## 2 Search methodology

**Figure 2:**
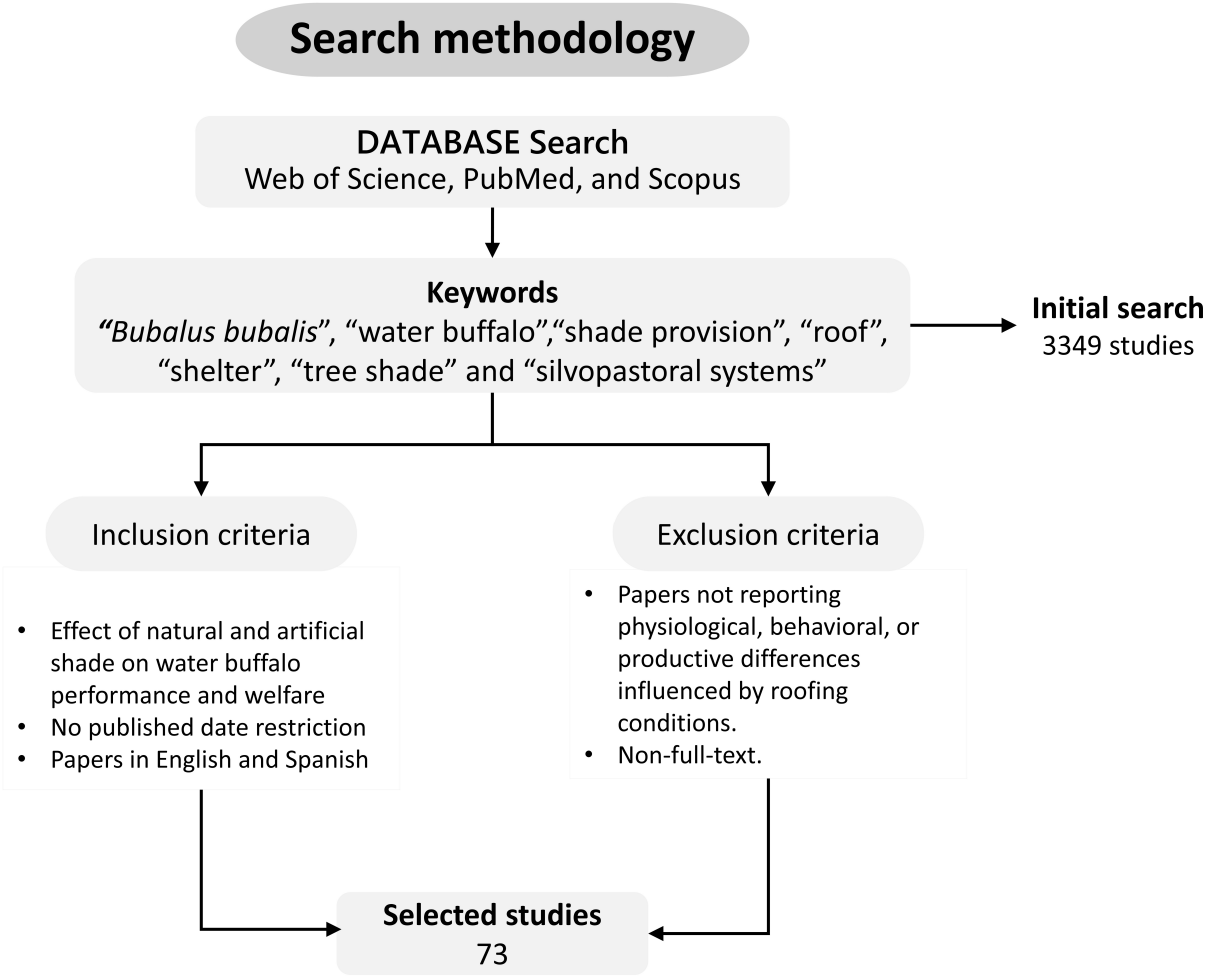
Search strategy, criteria, and selection process for the review.

A comprehensive literature search was conducted on the Web of Science, PubMed, and Scopus databases. The search was performed by three authors of the present review. The following keywords were used in combination to find publications available until October 8, 2025 (a period of approximately 40 years), using “OR” and “AND” operators: “*Bubalus bubalis*,” “water buffalo,” “shade provision,” “roof,” “shelter,” “tree shade,” and “silvopastoral systems.” Included studies assessed the effect of artificial or natural shade on the behavior, physiology, and productive performance of both water buffalo (*Bubalus bubalis*) and cattle (*Bos taurus/indicus*). No published date restriction or language was established to include all available studies. Excluded papers were those that did not report differences according to the roofing conditions and non-full-text papers. The initial search retrieved 3,349 articles. After selecting the studies according to the criteria, 73 studies were retained for the final review. Figure 2 summarizes the search methodology.

## 3 Advantages of providing natural shade to water buffaloes

**Figure 3:**
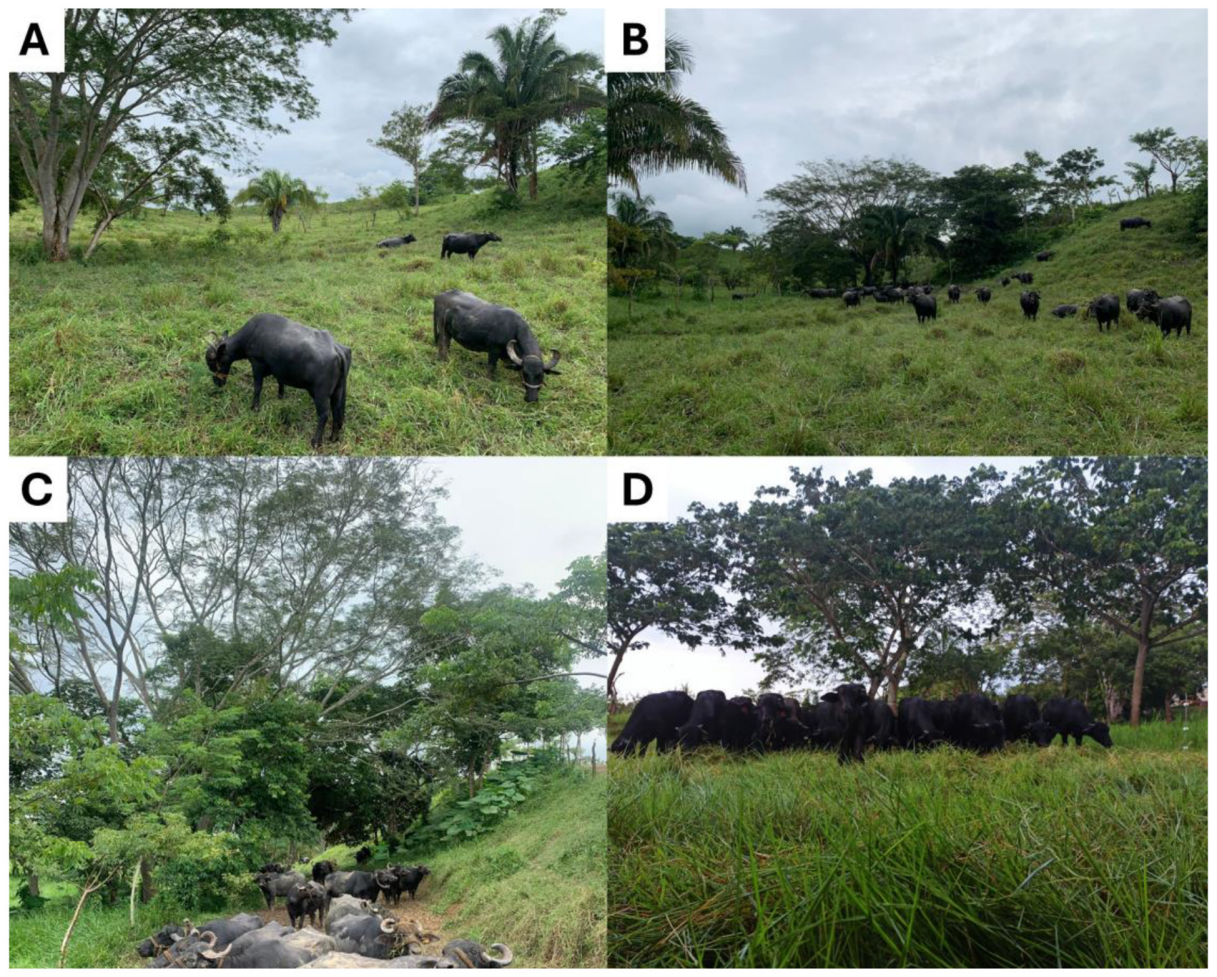
Water buffaloes in a SPS of the Mexican humid tropics. Natural shade is provided by native plants of the region, predominantly *Enterolobium cyclocarpum* **(A, B, D)**, *Melia azedarach* **(C)**, and *Syagrus romanzoffiana* **(A, B)** as the dominant shade tree. Photos taken by the authors.

Natural shade is an essential resource for mitigating the impact of HS on cattle and buffalo by reducing direct solar radiation and moderating the microclimate temperature (Figure 3) (29–34). Studies have shown that ruminants are highly motivated to seek shade. For example, lactating Jersey cows housed during the summer in pastures with shaded (5 m^2^/animal) and sunny (33 m^2^/animal) areas, increased shade use (by an average of 8.5% per hour during the morning until 12:50) and time spent in the shade (70% by area) (35). Similarly, Holstein and Jersey × Holstein cross-bred dairy heifers stayed longer in the shade during the summer (137 ± 36 min/day) than in autumn (88 ± 50 min/day). This is associated with the influence of shade on microclimate characteristics. For instance, shaded areas can have an environmental temperature that is 7.3 °C lower than non-shaded areas (36).

Provision of natural shaded shelters has several physiological benefits in ruminants by preventing alterations associated with HS, which directly reflects the animals' ability to maintain thermal balance (37, 38). For example, Garcia et al. (39) reported that dairy buffaloes kept in a SPS with tree shade had lower heart rate (HR) and RT compared to those without access to shade, demonstrating the effectiveness of natural shade in maintaining thermoregulation. This has been consistently observed in buffaloes raised under high THI conditions, where strong positive correlations between environmental variables (temperature and THI) and physiological responses such as RR, HR, and RT were reported (37, 40).

In particular, increases in RR are considered a reliable indicator of heat load in ruminants (41). However, the intensity of this compensatory response differs according to the region and breed. For instance, in crossbred Murrah/Mediterranean buffaloes, a THI ranging from 72.45 to 87.26 increased RR to up to 41.04 mov/min at 12:00 h (42), a value significantly higher than the increase observed in water buffaloes in Soure, Pará, Brazil (of 23.1 bpm) (37). Comparable results have also been observed in cattle under similar climatic conditions (43). This has also been registered in lactating Holstein cows reared under climatic conditions averaging 29.5 °C (38). In these animals, non-shaded cows had a significant increase in RR (>66.13 times/min), in contrast with shaded animals (62.07 times/min). This suggests that, regardless of the species, respiratory evaporation is a compensatory response to high environmental temperatures.

Providing shaded areas has also been shown to influence the animal RT, as observed in Murrah/Mediterranean buffaloes reared in a hot and humid tropical climate during different thermal conditions and day hours (6:00–19:00 h) (42). The highest RT values (39.01 °C) were recorded at 15:00 h, when the solar radiation level reached values close to the maximum (2,443.1 MJ/m^−2^), exceeding the normal physiological range described for buffaloes (37.4–37.9 °C), and this increase was statistically significant. In another study, Athaíde et al. (37) found that buffaloes without shade, at the hottest time of the day (14:00), recorded the highest RT (39.1 ± 0.59 vs. 38.1 ± 0.45 °C). This is similar to what was reported in tropical cattle. Reis et al. (44) reported significant differences in the RT of Gyr dairy and Girolando cows with and without shade, maintaining RT within physiological ranges ( ≤ 38.5 °C). In contrast, Martins et al. (45) found that RT remained practically the same in Gyr cows, regardless of shade provision, ranging from 38.0 to 38.7 °C in the rainy season and 37.7–38.6 °C in the dry season. These findings suggest that, in some cases, *Bos indicus* breeds and their crossbreds may be able to maintain body temperature within physiological limits through their own regulatory mechanisms, although shade still contributes to improved comfort. However, this response is not consistently observed in *Bos taurus* cattle (43, 46).

Changes in RT are associated with central and peripheral thermoregulatory mechanisms (47). Thus, some studies have adopted measuring surface infrared temperatures, through infrared thermography (IRT), as a non-invasive tool for assessing thermal status in ruminants (7, 42, 48). An example is the study by Brcko et al. (42), where IRT was used to evaluate the response of Murrah/Mediterranean buffaloes reared without shade. During the hottest time of the day (at 13:50 and 14:23 h), surface ocular, cheek, back, and rump temperatures reached values of 36.49, 36.6, 38.71 °C, and 33.81 °C, respectively. In particular, ocular and cheek values recorded the highest correlation with RT (*r* = 0.929 and *r* = 0.922), and these associations were statistically significant, suggesting them as reliable thermal windows to assess HS in these hot temperatures. Moreover, when comparing the surface thermal response (in the udder, rump, and periocular region) of Gyr dairy cows housed with and without shade, Martins et al. (45) found a significant reduction of the surface temperature in cows provided with shade, by 1.0–1.8 °C. Tree leaves absorb, scatter, and insulate infrared radiation more effectively (34) than many artificial materials, preventing the artificial structure from heating up and re-emitting that thermal energy toward the animal and the soil.

Thermoregulation relies on both physiological and behavioral modifications. In this sense, ruminants modify their behavior according to the environmental characteristics. Available evidence suggests that access to natural shade in SPS consistently influences the expression of key ruminant behaviors, modulating both feeding (grazing and browsing) and thermoregulatory behaviors (shade-seeking, rumination, and idleness), in addition to reducing the need for compensatory strategies such as wallowing. These patterns have been recognized as sensitive indicators of HS (32, 37, 49, 50). Furthermore, in dairy cattle, natural shade can influence social dynamics by reducing competition for limited heat mitigation resources, which contributes to improved group stability and welfare (43, 46). Recent studies have used precision tools to quantify the level of behavioral agitation of Black Angus steers in response to heat stress. In this regard, Idris et al. (51) reported that, by using video-digital analysis, increases in heat load (average up to 33 °C) were accompanied by significant increases in the digitized movement of animals (350,000 pixel changes/5 min). This is directly related to discomfort behaviors such as scratching and, especially, constant stepping while standing.

**Figure 4:**
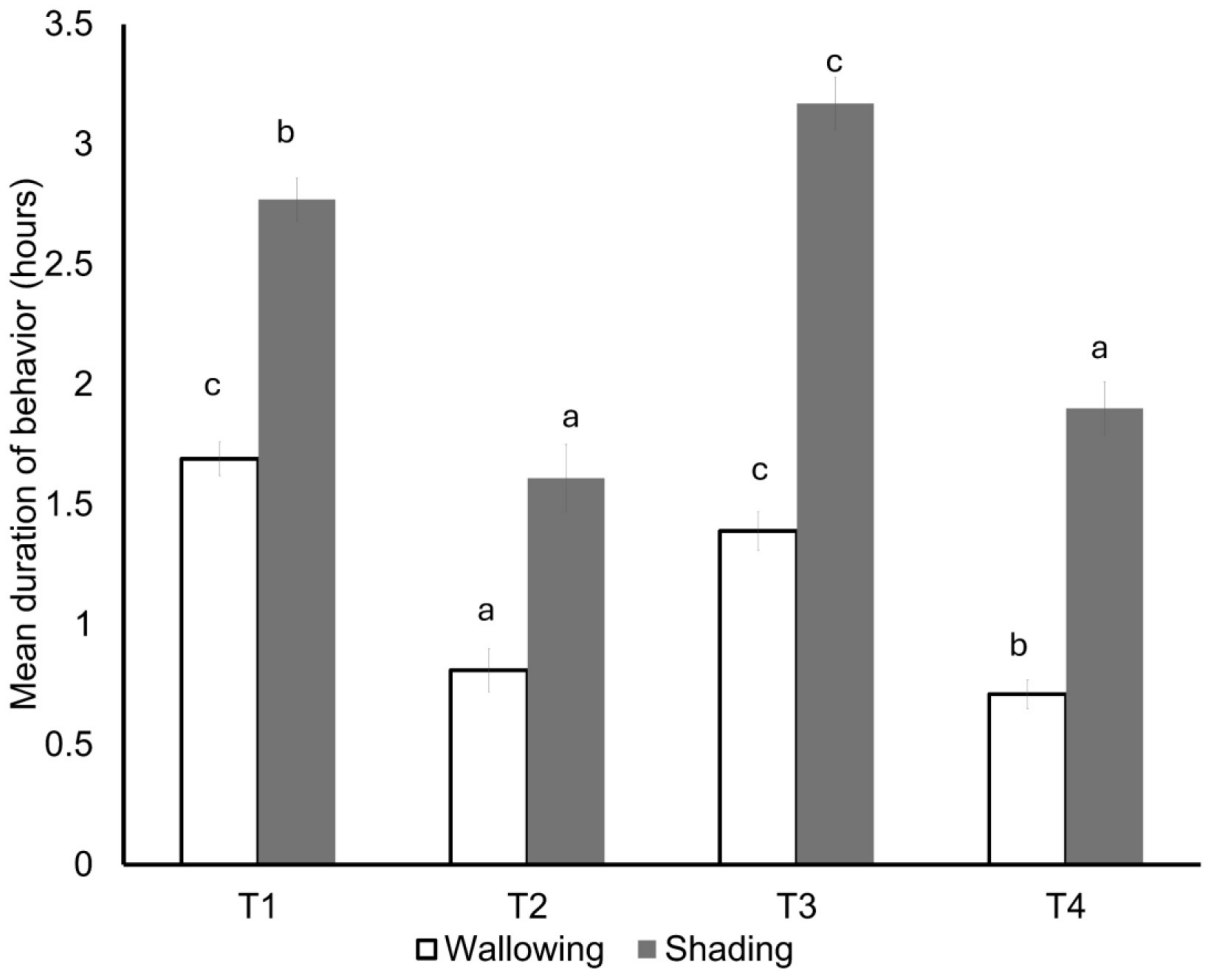
Thermoregulatory behavior of heifer water buffaloes exposed to four different environments. T1 (treeless system in intense HS), T2 (treeless system in moderate HS), T3 (SPS in intense HS), and T4 (SPS in moderate HS). Different letters above each bar represent statistically significant differences in wallowing or shade-seeking behavior between the four environments. This figure was prepared using information from Galloso-Hernández et al. (32).

In this regard, Galloso-Hernández et al. (32) evaluated the effect of SPS on the behavior of young water buffaloes under moderate and intense HS conditions. The authors compared four groups: T1 (treeless system in intense HS), T2 (treeless system in moderate HS), T3 (SPS in intenseHS), and T4 (SPS in moderate HS). The results can be observed in Figure 4, which shows the mean duration of thermoregulatory behaviors over 3 days, such as wallowing and shade-seeking. No significant differences were shown in wallowing time in the treeless group (T1 = 1.69 h) than in the silvopastoral group (T3 = 1.39 h), while shade seeking was significantly higher in buffalo with access to trees (T3 = 3.17 h) compared to those in full sun (T1 = 2.77 h). Additionally, the authors reported a significant decrease in grazing time in these groups (32).

**Figure 5:**
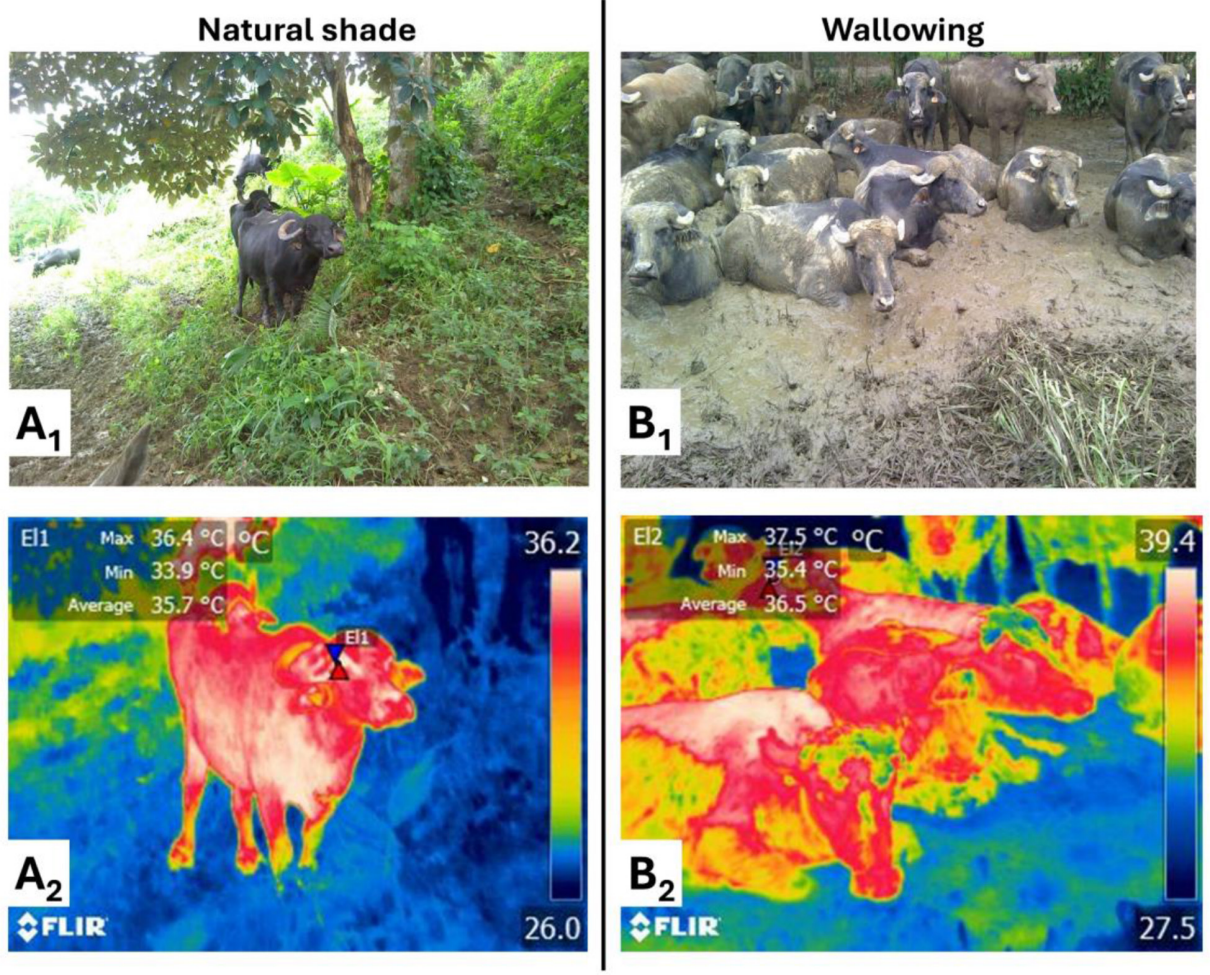
Effect of natural shade and wallows on periocular temperature in water buffalo at noon. **(A**_**1**_**)** Digital image of water buffaloes housed under natural shade with *Paspalum fasciculatum, Hymenachne amplexicaulis*, and *Enterolobium cyclocarpum* trees. **(A**_**2**_**)** Thermal images delimiting the periocular thermal window, where shaded buffaloes reached a maximum periocular surface temperature of 36.4 °C and a minimum of 33.9 °C (average 35.7 °C). **(B**_**1**_**)** Digital image of water buffalo wallowing. **(B**_**2**_**)** Wallowing buffaloes recorded a maximum, minimum, and average periocular surface temperature of 37.5, 35.4, and 36.5 °C, respectively. Thermal images were analyzed using the rainbow palette.

Another study compared a conventional system (only pasture without trees) to a silvopastoral one (pastures combined with trees) (50). The results showed that buffaloes spent significantly more time grazing in systems with natural shade (7.49 vs. 5.96 h), while wallowing time was significantly lower in SPS (1.18 vs. 2.35 h). Additionally, buffaloes in SPS under moderate HS had a significantly higher number of animals standing (4.07 ± 0.50 of a total of nine buffaloes) and for longer (3.66 h in 36 h) compared to conventional systems under moderate HS (3.41 ± 0.51 and 3.06 h, respectively). Particularly, standing is considered a compensatory behavioral response of cattle when exposed to high environmental temperatures. A standing posture facilitates convective heat transfer by exposing a larger body surface area to the air (52). Preliminary studies by the authors of this review reported that the periocular temperature in buffaloes housed under natural shade was 1.8 °C lower compared to that when animals perform wallowing, as shown in Figure 5.

These results show that natural shade in SPS significantly modulates buffalo behavior under HS conditions. These systems also promote longer periods of active postures, such as standing, reflecting a better balance between thermal comfort and feeding (32, 50). Moreover, evidence from dairy cattle indicates that shaded environments are associated with more stable social interactions and fewer agonistic events, likely due to lower thermal discomfort and reduced competition (43, 46). However, similar studies specifically evaluating social behavior in water buffaloes are still lacking, and this aspect warrants further investigation.

Dos Santos et al. (53) also evaluated the behavioral response to access to shade of Murrah buffaloes in a SPS in the Eastern Amazon. The ambient temperature was significantly lower (*p* < 0.05) in the shaded areas compared to those exposed to the sun. Grazing time decreased significantly during the hours of greatest solar radiation, while the percentage of buffaloes ruminating in a lying position under the shade between 10 am and 1:55 pm was higher (14.42%) than in non-shaded animals (1.12%). Likewise, buffaloes spent significantly more time in inactivity in the shade between 10 am and 1:55 pm (22.42% lying down and 17.81% standing) compared to the non-shaded areas during the same period of the day (1.60 and 1.60%, respectively; *p* < 0.05). Overall, female buffaloes spent more time in the shade during critical hours of the day, reducing grazing and increasing rumination and inactivity as thermoregulation strategies. Athaíde et al. (37) also concluded that shaded Murrah buffaloes spent significantly more daily time grazing (629.5 min) than unshaded animals (518.2 min). In the same study, daily idleness was significantly lower in shaded than in non-shaded animals (367.0 vs. 579.2 min). These results demonstrate that the availability of trees not only offers protection from direct sunlight but also stimulates increased feeding activity.

The integration of trees into cattle production systems not only contributes to mitigating HS but can also has direct implications on the productive and reproductive efficiency of animals. Although research in buffaloes is limited, studies performed in cattle have reported significant benefits for the productive performance of animals. In this sense, Van laer et al. (54) evaluated the milk yield of Holstein–Friesian dairy cows reared with and without shade (young trees combined with black shade cloths) on pasture conditions. The results showed that daily milk yield without access to shade decreased significantly by 1.0/day when heat load indexes, a more accurate indicator than THI by integrating solar radiation and wind speed, increased from 65 to 85 (25.1 to 24.1 L/day), whereas in shaded cows, the yield remained without significant changes. Productive benefits were recorded by Assani Seidou et al. (55), in a study that compared the performance of dairy cows housed in four systems: traditional silvopasture (TSS), improved Silvopasture (ISS), small-scale agrosilvopasture (SIAS), and large integrated agrosilvopasture (LIAS). The results showed that the ISS (which integrated trees such as *Khaya senegalensis* and *Leucaena leucocephala*) recorded the statistically significant highest milk yield (2.8 ± 0.63 kg/day/cow), in contrast to TSS, SIAS, and LIAS (1.0 ± 0.18, 1.2 ± 0.26, and 1.8 ± 0.61, respectively).

Some other studies have reported no influence of shade provision on milk yield, but an influence on milk characteristics. For example, Abreu et al. (56) found that, although daily milk yield between shaded and non-shaded cows did not significantly differ (19.4 vs. 17.7 kg/d), the milk from cows with shade had significantly higher protein concentration (3.0 vs. 2.8 g/100 g) and density (1,029.6 vs. 1,028.3 g/L). Martins et al. (45) did not report significant differences regarding milk yield of lactating and non-pregnant *Bos Indicus* Gyr cows exposed to full sun vs. natural shade from *Eucalyptus urophylla* × *Eucalyptus urograndis* trees. However, when compared to non-shaded cows, the authors found that shaded cows had a significantly higher number of ovarian follicles (+5.32 ovarian follicles), a higher number of total oocytes (+4.91 oocytes), and a higher number of viable oocytes (+2.82 oocytes). Additionally, the number of transferable embryos per cow was 2.27 ± 1.7 in shade vs. 0.55 ± 0.28 in the sun (+1.72 embryos; *p* = 0.0017) (45).

Although studies directly evaluating productive responses to natural shade in buffaloes are scarce, some evidence suggests similar potential benefits. For example, García et al. (39) reported that buffaloes in SPS maintained lower physiological stress and more stable feeding behavior compared to those without shade. Athaíde et al. (37) observed that shaded buffaloes spent more time grazing and less time idle, indicating greater opportunity for nutrient intake, which could positively influence weight gain and milk yield. However, long-term studies evaluating parameters such as daily milk yield, growth rates, or reproductive performance in buffaloes under natural shade are still limited, indicating a relevant gap in the literature.

These findings suggest that providing production systems with trees as natural shade sources has a favorable effect on the animals' microclimate conditions, and also influences physiological, behavioral, and potentially productive responses of ruminants, which may lead to improvements in performance and reproductive efficiency (Table 1). Nevertheless, more species-specific research in buffaloes is necessary to confirm these trends and to better quantify the magnitude of the productive benefits derived from natural shade.

**Table 1:** Studies addressing the effect of natural shade on the physiology, behavior, and productive performance of water buffalo and cattle.

**Breed/species**	**Shade type**	**Environmental conditions**	**Parameters**	**Outcomes**	**References**
Jersey *(Bos taurus)*	SPS with a shaded area of 5 m^2^/animal	Solar radiation 727 W/m^2^	•Shade use	•↑ Shade use	Deniz et al. (35)
Holstein and Jersey × Holstein cross-bred *(Bos taurus)*	*Eucalyptus* sp. trees (~20 m^2^/animal)	Ambient temperature (summer) 34.0 ± 0.45 °C	•Environmental temperature •Time in the shade	•↓ Environmental temperature •↑ Time in the shade	Cardoso et al. (36)
Murrah *(Bubalus bubalis)*	Cueira (*Crescentia cujete*) native trees	Ambient temperature 34.9 °C and THI of 85	•THI •RR •RT •IRT (eyes, eat, tail insertion, vulva) •Idleness •Grazing •Ruminating	•↓ THI •↓ RR •↓ RT •↓ IRT temperatures •↓ Idleness •↑ Grazing •↑ Ruminating	Athaíde et al. (37)
Holstein *(Bos taurus)*	Shade of poplar trees	Ambient temperature 29.5 °C Humidity 80% Solar radiation 873 W/m^2^ d	•RR •RT	•↓ RR •↓ RT	Li et al. (38)
Murrah × Mediterranean (*Bubalus bubalis)*	Pens with *Brachiaria brizantha* (cv. Marandu) grass	Air temperature 23.14–37.28 °C THI 72.45–87.26 Solar radiation 2,443.1 MJ m^−2^	•IRT	•IRT temperatures in the eye and cheek aid in evaluating HD	Brcko et al. (42)
Gyr and Girolando (*Bos indicus*)	*Eucalyptus urograndis*	Air temperature 24 ± 0.7–31.5 ± 0.9 °C THI 79–84	•RT	•↓ RT	Reis et al. (44)
Gyr (*Bos indicus)*	•Direct solar radiation •Natural shade (*Eucalyptus)*	Solar radiation 542.7 MJ/m^−2^ and 306.7 MJ/m^−2^	•RT •IRT (udder, rump, and periocular region) •Milk yield	Natural shade: •– RT •↓ IRT temperatures •– Milk yield	Martins et al. (45)
Heifer buffaloes (*Bubalus bubalis)*	•Conventional system •SPS	THI < 75 or >75	•Shade seeking •Wallowing	SPS: •↓ Shade seeking •↓ Wallowing	Galloso-Hernández et al. (32)
Heifer buffaloes *(Bubalus bubalis)*	SPS with scattered trees	THI < 75	•Grazing •Wallowing	•↑ Grazing •↓ Wallowing	Galloso-Hernández et al. (50)
Murrah (*Bubalus bubalis)*	SPS	Ambient temperature 32.57–33.80 °C	•Ambient temperature •Grazing time •Rumination •Idling •Standing	•↓Ambient temperature •↑ Grazing •↑ Rumination •↑ Idling •↑ Standing	Dos Santos et al. (53)
Holstein–Friesian cows *(Bos taurus)*	Mixed system of young trees combined with black shade cloths (80%)	HLI 50.8–85.5 Ambient temperature 16.0–30.2 °C	•Milk yield	•↑ Milk yield	Van laer et al. (54)
Borgou, Gudalii, White Fulani and Crossbreed (*Bos taurus and Bos indicus)*	*Khaya senegalensis* and *Leucaena leucocephala*	N/S	•Milk yield	•↑ Milk yield	Assani Seidou et al. (55)
Holstein (*Bos taurus)*	•Non-shaded •Natural tree shade	Air temperature 25.9 ± 2.7–28.4 ± 3.1 THI 76 ± 3.4	•Milk yield •Milk protein •Milk density	•↑ Milk yield •↑ Protein •↑ Density	Abreu et al. (56)

IRT, infrared thermography; N/S, not specified; RR, respiratory rate; RT, rectal temperature; THI, temperature-humidity index; HLI, head load indexes; SPS, silvopastoral system; ↑, significant increase; ↓, significant decrease; –, no effect.

## 4 Advantages of artificial shade in water buffalo farming and comparative insights from dairy cattle

**Figure 6:**
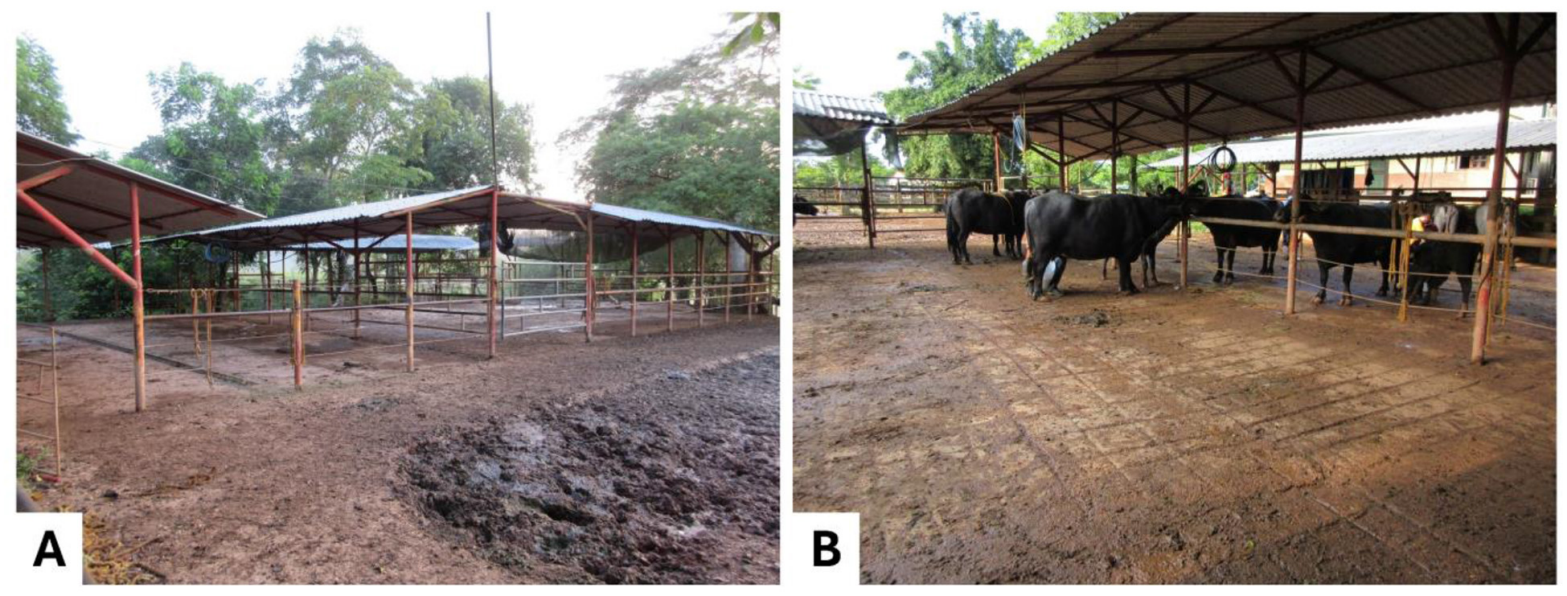
Artificial shade in buffalo farming systems. **(A)** A shade cloth is also used to provide artificial shade to Buffalypso buffalo dairy cows in the humid tropics of Mexico. **(B)** A galvanized sheet roof can be observed. Photos taken by the authors.

In buffalo farms, shelters and roofed open areas are alternatives even when animals are provided with natural shade (57) (Figure 6).

Some studies have compared the effect that shade and non-shade environments have on animals' physiology. For example, Gu et al. (9) determined that a loafing area with a shade shelter reduces air temperature by 1.5 °C and the RR of Dehong buffalo calves (25.3–26.1 breaths/min) when compared to non-shaded animals (28.5–31.6 breaths/min). In other studies, such as the PhD thesis by Barman (57), four roofing materials were compared to determine their effect on RT and RR of buffalo calves. The author compared asbestos (T1), pre-painted Corrugated Galvanized Iron sheet (T2), polythene (T3), and galvanized iron (T4). When comparing winter, summer, and rainy seasons, the highest THI was recorded at 14:00 h during summer (86.92 ± 0.82), a value with a high risk of HS. During this season, the overall maximum ambient temperature was significantly lower in T3 (33.34 ± 0.41 °C) than in T1 (35.75 ± 0.56 °C), T2 (36.18 ± 0.33 °C), and T4 (36.72 ± 0.26 °C). Relative humidity values were also significantly lower in polythene roofs (T3, up to 63.88 ± 1.45%) when compared to T1 (74.55 ± 1.98%), T2 (76.54 ± 1.65%), and T4 (77.20 ± 1.38%). This is attributed to the high thermal absorption of polythene, retaining heat without becoming saturated. Therefore, it raises the temperature of the surrounding air and promotes the dispersion of moisture. Regarding physiological variables, polythene roofs were associated with lower RR (35.00 ± 1.16 breaths/min), while the highest value was recorded with galvanized roofs (43.40 ± 1.69 breaths/min). The lowest RT was recorded for T1 (39.10 ± 0.04 °C) while the highest was for T4 (39.71 ± 0.04 °C). Other studies have also evaluated the effect of eight roof materials on the RR of Egyptian water buffalo in Nile Valley farms (58). The authors compared roofs made with palm leaves, wood vein with cotton wood and rice straw, sheet metals, asbestos, wood and canvas, cement, mat, and maize stoke. The highest and statistically significant RR was recorded in animals housed under an asbestos roof (35.00 ± 0.22 breaths/min), while the significantly lowest RR was observed in the maize stoke group (17.09 ± 0.75 breaths/min).

The influence that roof material has on physiological parameters such as RR is associated with the cardio-respiratory response of ruminants to heat stress HS (59). Khongdee et al. (24) also evaluated the physiological response (RT and plasma cortisol values) of male buffaloes sheltered under normal roofs (corrugated iron) and those covered with polypropylene shade cloth (corrugated iron + shade cloth with 80% shade factor). Ambient variables such as THI inside the shed at 14:00 revealed a significantly lower index in the modified roof condition (91.95 ± 0.44) than under normal sheds (93.66 ± 0.30). In addition, buffaloes housed in the shade cloth group significantly reduced their overall RT assessed over 56 days (39.14 ± 0.07 vs. 40.00 ± 0.10 °C) and cortisol concentrations (2.14 ± 0.24 vs. 3.38 ± 0.37 ng/ml). Cortisol is a physiological marker of stress (60), its concentrations have been reported to increase in dairy cattle exposed to HS (THI up to 86.7) (61). For example, in thermotolerant Holstein cattle exposed to HS (THI >80), cortisol concentrations were significantly lower (8.33 ± 33.30 ng/ml) than in thermosensitive cows (89.28 ± 51.28 ng/ml), showing the association of cortisol with HS (62). Thus, roof modifications reduce heat load.

**Figure 7:**
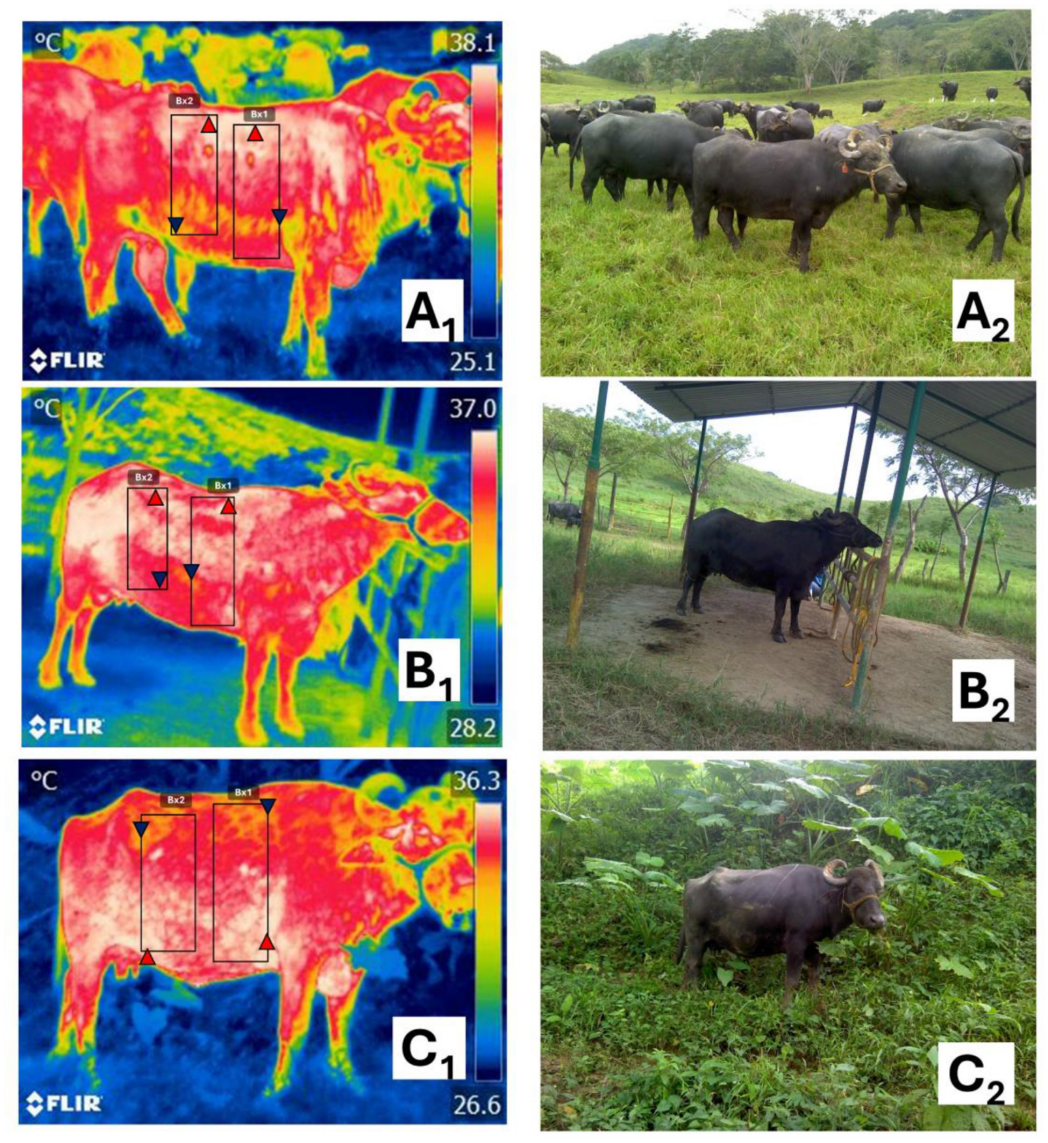
Thermal and digital images of water buffalo in the humid tropics under three environmental conditions: **(A**_**1**_**, A**_**2**_**)** direct solar radiation, **(B**_**1**_**, B**_**2**_**)** artificial shade, and **(C**_**1**_**, C**_**2**_**)** natural shade. Thermal differences are observed in the animals in the two thermal windows among the three environmental conditions. For the coastal region **(B**_**1**_**)**, the highest maximum, minimum, and average values were recorded in condition **(A**_**1**_**)**, being 38.3, 35.7, and 36.3 °C, respectively. Under shaded areas, animals kept under natural shade **(C**_**1**_**)** recorded the lowest maximum (36.7 °C), minimum (33.6 °C), and average (35.2 °C) values, in contrast with buffaloes under artificial shade **(B**_**1**_**)**, who recorded 37.1, 34.6, and 36.0 °C, respectively. When analyzing the records for the cranial abdominal region **(B**_**2**_**)**, similar to the coastal region, animals under natural shade recorded the lowest maximum (36.5 °C), minimum (33.7 °C), and average (35.3 °C) values. In contrast, the highest temperatures were recorded in **(A**_**1**_**)** (38.2, 33.4, and 35.9 °C, respectively), followed by **(B**_**1**_**)** (37.2 °C, 34.2 °C, and 35.8 °C, respectively).

When assessing milk cortisol levels, Choudhary et al. (63) evaluated the influence of heat mitigation strategies (including a group with trees plus an asbestos roof) when exposing lactating Murrah buffaloes to HS. Animals housed in open areas without shade showed the highest milk cortisol values (61.46 ng/ml), in contrast to buffaloes with shade, where cortisol levels were significantly reduced by 11.80%. In the same study, the authors adopted IRT to assess the surface temperature of buffaloes. Considering the thermal values for the muzzle, eye, and forehead, the group kept in the open area had significantly higher temperatures (43.58 ± 0.21, 44.09 ± 0.19, and 47.30 ± 0.36 °C, respectively) than asbestos-roofed buffaloes (37.53 ± 0.88, 41.58 ± 0.34, and 42.35 ± 0.26 °C, respectively). Changes in surface temperature directly reflect central body temperature (e.g., RT) (64–66). The thermal response has also been evaluated by the authors of the present review. Preliminary results are shown in Figure 7, where the surface thermal response of water buffaloes was evaluated under three conditions: direct solar radiation, artificial shade, and natural shade.

Other authors have highlighted the benefits of providing a roofed area plus mist fans or showers (11, 67). In Younas et al.'s (68) study, a control group with only a roof and groups with additional elements such as ceiling fans and showers were compared to evaluate the physiological adaptations of Nili-Ravi buffaloes during the summer. It was found that animals in the control group recorded the significantly highest RT (101.12 ± 0.06°F), pulse rate (50.6 ± 0.90 pulses/min), RR (19.0 ± 0.31 breaths/min), and IRT temperatures in the mid back region (29.8 ± 0.69 °C) in comparison with a group housed under a roof with ceiling fans and showers (100.08 ± 0.07 °C, 44.8 ± 0.51 pulses/min, 16.6 ± 0.21 breaths/min, and 26.3 ± 0.55 °C, respectively). Comparably, in lactating Nili-Ravi buffaloes housed under shade (control) or provided with additional fans and sprinklers (69), animals kept only under shade had the highest RT (101.69°F) in comparison with the animals housed with shade + sprinklers + fans (100.85°F). This indicates significant differences due to the combination of cooling strategies increasing the efficacy in preventing HS.

Ashour et al. (22) found that exposing buffalo calves to HS (ambient temperature of 40 °C) significantly decreased feed intake (animals had a daily intake of concentrate of 1.11 ± 0.09 kg/h/day), while daily drinking increased by 20%. In the study, forage availability was not the factor that caused weight loss, since both forage and concentrate were provided *ad libitum*. The decrease in feed intake is elicited by the inhibitory action of HS on the lateral hypothalamic appetite center (23) and directly affects the productive performance of buffalo farms (as discussed later). However, in tropical regions, managing grazing schedules should be considered an adaptive strategy, since grazing mainly occurs during the morning, late afternoon, and part of the night to avoid the hours of highest heat load (32). Under these free-range grazing conditions, modifying the behavioral pattern acts as a mitigation strategy that reduces the negative impact of high temperatures on productive performance.

Dry matter intake (oat grass silage) was also evaluated in Nili-Ravi buffaloes allocated to either a shade group (control) or those with shade + sprinklers +fans (69). The highest dry matter intake (90%) of the total feed was recorded in the second group, with 14.61 ± 1.18 kg/day. In contrast, control animals had a lower intake (75% of the total), with 12.45 ± 0.39 kg/day. Moreover, water intake increased in control animals when compared to the shade + sprinklers + fan group (108.29 ± 3.95 vs. 95.04 ± 1.8 liters/day). Decreasing dry matter intake can be considered a strategy to reduce heat production associated with metabolic processes. In another study, the effect of shelters with corrugated iron roofs and those with corrugated iron+polypropylene cloth was compared in male buffaloes (24). The results showed that behaviors such as daily water intake were significantly lowered by 5 L in animals in the polypropylene cloth group (29.71 ± 0.86 liters/head/day). In the same group, roughage intake was significantly higher (6.44 ± 0.19 kg/head/day). Notably, in Murrah × Mediterranean buffaloes, providing shade combined with access to water for immersion increased lying and grazing time compared with shade alone, indicating additive benefits of artificial shade with complementary cooling resources (40). These findings are consistent with longitudinal observations in a mixed-resource paddock (natural shade, artificial shade, ponds), where buffalo cows increased immersion time markedly during hotter months (up to ~43% of the diurnal window) and immersion time tracked temperature/THI, reinforcing that water access is not redundant but complementary to shade in real-world grazing layouts (70).

In calves housed in a loafing area with shelter, behavioral outcomes showed that ambient temperatures above 30 °C significantly increased shade use by 41.4%, and significantly lowered the time standing (20.7 ± 4.4 vs. 33.2 ± 3.3%) and lying (37.8 ± 10.4 vs. 56.7 ± 2.7%) during the 2 months of observation (9). These differences are related to the need for animals to dissipate heat through a standing or lying position to lose heat by convection or conduction, respectively (71). In this sense, HS in dairy cows is associated with increased standing time, panting, drinking, and reduced feed intake (11, 72, 73). This is related to what Ximenes et al. (40) reported in Murrah × Mediterranean buffalo calves, heifers, and cows assigned to either only shade or shade plus wallowing water groups. While no physiological differences were observed in RT, RR, and HR, lying and grazing time significantly increased in the shade+wallowing group (up to 96.23 and 308.48 min, respectively) during the hot season (December to April).

As feeding and grazing times are altered in ruminants during HS, this directly influences productive parameters (e.g., milk yield, average daily weight gain) (72). For example, in buffalo calves, exposure to ambient temperatures around 40 °C resulted in a significant and average daily loss of weight of 0.49 ± 0.03 kg (22). The same study shows that providing shelters with artificial shade

reduces the impact of HS on the productive traits. However, the roof material also influences the animals' performance through differences in thermal insulation. In this sense, Omran et al. (58) compared eight different roofing materials to determine their effect on the total milk yield of Egyptian buffaloes. It was found that milk yield was significantly higher in buffaloes under mat roofs (2,135 ± 24.57 kg), increasing milk yield by three-fold. Animals under the mat roof also recorded the longest lactation period (225 ± 1.89 days), outperforming the other groups. Maize stoke roof was the second most effective option, recording significantly increased milk yield and lactation period (1,732 ± 71.13 kg and 211 ± 4.06 days, respectively) when compared with industrial materials. In contrast, asbestos roof drastically affected the productive performance of animals by significantly reducing both milk yield and lactation period (632.5 ± 30.00 kg and 133 ± 4.23 days, respectively). These results suggest that natural materials or those with high insulation capacity provide a superior microclimate for thermoregulation, while highly reflective or heat-retaining materials, such as asbestos, might affect productivity, causing a decrease of up to approximately 70% in milk yield compared to mat roofs.

Additional strategies, such as sprinklers, also influence milk yield. In lactating Nili-Ravi buffaloes receiving oat grass silage with 41% of dry matter during the hot-dry season (ambient temperature up to 33.90 °C), Ahmad et al. (69) found that providing only shade is insufficient to prevent the consequences of HS and recommend additional mitigating strategies. A significantly higher THI was observed in animals kept under shade only (81.1 ± 0.73), in contrast to those housed with shade, sprinklers, and fans (THI of 77.69 ± 0.47). Moreover, milk production was highly influenced by the environment, significantly decreasing to 5.60 ± 1.19 kg/day in the shade-only group. Conversely, milk yield significantly increased to 8.74 ± 1.15 kg/day when providing shade, sprinklers, and fans.

The studies discussed and summarized in Table 2 suggest that providing shade could be a suitable technique to mitigate the physiological and behavioral alterations elicited by HS. Furthermore, including resources such as sprinklers or fans plus shade and water for immersion in combination with artificial shade is a potential alternative to prevent HS in water buffalo, which might improve productive outcomes under hot conditions. Beyond shading *per se*, water immersion is suggested as an effective cooling avenue for buffaloes under heat load. Controlled comparisons tend to show higher lying and grazing with shade + water vs. shade only (40), while longitudinal field data mentions a seasonal scaling of immersion time with thermal load, independent of drinking frequency (70). Together, these lines of evidence suggest that designing shaded areas in tandem with accessible ponds/pools can help mitigate the physiological and behavioral effects caused by HS.

**Table 2:** Studies on the effect of artificial shade on physiology, behavior, and productive performance of water buffalo and cattle.

**Breed/species**	**Roof material**	**Environmental conditions**	**Parameters**	**Outcomes**	**References**
Nili-Ravi buffalo (*Bubalus bubalis*)	•Normal roof •Roof + sprinklers + fans	THI 81.1 ± 0.73 and 77.69 ± 0.47	•THI •RT •Dry matter intake •Water intake •Milk yield	Roof + sprinklers + fans: •↓ THI •↓ RT •↑ Dry matter intake •↓ Water intake •↑ Milk yield	Ahmad et al. (69)
Buffalo (*Bubalus bubalis*)	•Asbestos (T1) •Pre-painted corrugated galvanized iron sheet (T2) •Polythene (T3) •Galvanized iron (T4)	Ambient temperature (maximum) and Relative humidity T1: 35.75 ± 0.56 °C and 74.55 ± 1.98% T2: 36.18± 0.33 °C and 76.54 ± 1.65% T3: 33.34 ± 0.41 °C and 63.88 ± 1.45% T4: 36.72 ± 0.26 °C and 77.20 ± 1.38%	•Ambient temperature •HR •RR •RT	Polythene: •↓ Ambient temperature •↓ HR •↓ RR •↓ RT	Barman (57)
Murrah buffalo (*Bubalus bubalis*)	Trees + asbestos roof	THI 82.29–84.62	•Milk cortisol •IRT temperature (muzzle, eye, forehead)	•↓ Milk cortisol •↓ IRT temperatures	Choudhary et al. (63)
Dehong calves (*Bubalus bubalis*)	Loafing area with shade shelter	Ambient temperatures above 30 °C	•Air temperature •Shade use •RR •Standing position •Lying position	•↓ Air temperature •↑ Shade use •↓ RR •↓ Standing •↓ Lying	Gu et al. (9)
Buffalo (*Bubalus bubalis)*	•Corrugated iron roof •Corrugated iron + polypropylene cloth with 80% shade factor	THI 93.66 ± 0.30 and 91.95 ± 0.44	•THI •RT •Plasma cortisol •Roughage intake •Water intake	Corrugated iron + polypropylene cloth: •↓ THI •↓ RT •↓ Plasma cortisol •↑ Roughage intake •↓ Water intake	Khongdee et al. (24)
Buffalo (*Bubalus bubalis)*	•Palm leaves •Wood veins + cotton wood + rice straw •Sheet metals •Asbestos •Wood and canvas •Cement •Mat •Maize stoke	THI: 87.45 ± 0.07	•RR •Total milk yield •Lactation period	Mat and maize roof: •↓ RR •↑ Total milk yield and lactation period	Omran et al. (58)
Murrah × Mediterranean (*Bubalus bubalis*)	•Only shade •Shade + wallowing	Highest ambient temperature 40 °C THI < 70	•RR •RT •HR •Grazing time •Lying time	Shade + wallowing: •– RR •– RT •– HR •↑ Grazing •↑ Lying	Ximenes et al. (40)
Nili-Ravi buffalo (*Bubalus bubalis*)	•Normal roof •Roof + ceiling fans + showers	THI 77–85 Environmental temperature 29.3–34.6 °C Humidity 51.3–54.6%	•RR •RT •Pulse rate •IRT temperature (mid back)	Roof + ceiling fans + showers: •↓ RR •↓ RT •↓ Pulse rate •↓ IRT temperature	Younas et al. (68)

IRT, infrared thermography; HR, heart rate; RR, respiratory rate; RT, rectal temperature; THI, temperature-humidity index; ↑, significant increase; ↓, significant decrease; –, no effect.

Providing natural and artificial shade to water buffaloes is an alternative to preventing the adverse effects linked to HS. It has been shown that, although water buffaloes are adapted to hot and humid climates, when exposed to direct sunlight, compensatory physiological responses arise to decrease body temperature. While buffaloes have natural thermoregulatory behaviors (i.e., wallowing), shade provision significantly contributes to maintaining body temperature within the thermoneutral zone by decreasing responses such as tachypnea. However, most of the studies addressing the effect of shade in bovines are focused on the *Bos taurus*/*Bos indicus* species. Therefore, further research is needed on water buffaloes, roof material, and their effect on animal health and performance.

## 5 Conclusion

Access to shade, whether natural or artificial, is a fundamental tool for mitigating the effects of HS in water buffalo, especially in tropical and subtropical zones, where environmental conditions challenge homeostasis. While available scientific evidence suggests that both natural and artificial shade reduce alterations in physiological parameters related to HS, such as increases in HR, RR, and RT. Shade provision also promotes behaviors associated with greater thermal comfort (e.g., increasing feeding and lying time). However, current evidence is predominantly limited to descriptive and temporal observations, lacking in-depth longitudinal analysis. Regarding artificial shade, when designing the shelter and roof materials and/or characteristics (e.g., dimensions, orientation, and radiation permeability), the anatomical differences of water buffalo must be considered due to their greater susceptibility to HS when compared to *Bos* cattle. Although less studied, the effects on metabolism, endocrine function, and reproduction suggest a potential positive impact on breeding efficiency and embryonic quality. Nonetheless, most of these findings still originate from studies in dairy cattle rather than buffaloes, highlighting a significant research gap regarding the effect of shade on water buffaloes. Research on water buffalo still needs to be expanded to include long-term evaluations of productive performance (e.g., milk yield, growth rate, feed efficiency, fertility), as well as the interaction between shade and other heat-mitigation resources such as fans, sprinklers, and water for immersion. Moreover, the economic viability and sustainability impact of shade-based strategies under different production systems and climatic conditions of water buffaloes is still under research. Analyses of the cost-benefit and sustainability of these strategies is required. This approach is essential for designing systems that provide shade to livestock, improving their welfare and adaptability to climate change, but also for quantifying its long-term impact on the overall efficiency of animal production.
